# Functions and Regulation of the PTEN Gene in Colorectal Cancer

**DOI:** 10.3389/fonc.2013.00326

**Published:** 2014-01-16

**Authors:** Francesca Molinari, Milo Frattini

**Affiliations:** ^1^Laboratory of Molecular Pathology, Institute of Pathology, Locarno, Switzerland

**Keywords:** PTEN, colorectal cancer, mutation, immunohistochemistry, prognosis, predictive, EGFR-targeted therapies

## Abstract

Phosphatase and TENsin homolog deleted on chromosome 10 (PTEN) is a tumor suppressor gene located at chromosome 10q23.31, encoding for a 403-amino acid protein that possesses both lipid and protein phosphatase activities. The main function of PTEN is to block the PI3K pathway by dephosphorylating phosphatidylinositol (PI) 3,4,5-triphosphate to PI-4,5-bisphosphate thus counteracting PI3K function. PTEN inactivation is a frequent event in many cancer types and can occur through various genetic alterations including point mutations, large chromosomal deletions, and epigenetic mechanisms. In colorectal cancer (CRC) PTEN is altered through mixed genetic/epigenetic mechanisms (typically: mutations and promoter hypermethylation or 10q23 LOH and promoter hypermethylation), which lead to the biallelic inactivation of the protein in 20–30% of cases. The role of PTEN as a prognostic and predictive factor in CRC has been addressed by relatively few works. This review is focused on the report and on the discussion of the studies investigating these aspects. Overall, at the moment, there are conflicting results and, therefore it has not been clarified whether PTEN might play a prognostic role in CRC. The same is valid also for the predictive role, leading to the fact that PTEN evaluation cannot be used in routinely diagnosis for the early identification of patients who might be addressed to the treatment with EGFR-targeted therapies, at odds with other genetic alterations belonging to EGFR-downstream pathways. The reason of discordant results may be attributable to several issues: (1) the size of the analyzed cohort, (2) patients inclusion criteria, (3) the methods of assessing PTEN alteration. In particular, there are no standardized methods to evaluate this marker, especially for immunohistochemistry, a technique suffering of intra and inter-observer variability due to the semi-quantitative character of such an analysis. In conclusion, much work, especially in large and homogeneous cohorts of cases from different laboratories, has to be done before the establishment of PTEN as prognostic or predictive marker in CRC.

## Introduction

**Figure 1:**
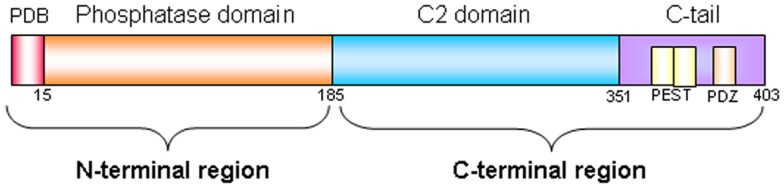
**PTEN protein structure**. PTEN is composed of 403-amino acids and contains: a N-terminal region of 185 aminoacids (1–185) composed by a PIP2-binding domain (PBD) and by a phosphatase domain of 218 aminoacid (186–403), and a C-terminal region composed by a C2 domain and by a C-terminal tail containing two PEST (proline, glutamic acid, serine, threonine) sequences, and a PDZ-interaction motif at the end.

**Figure 2:**
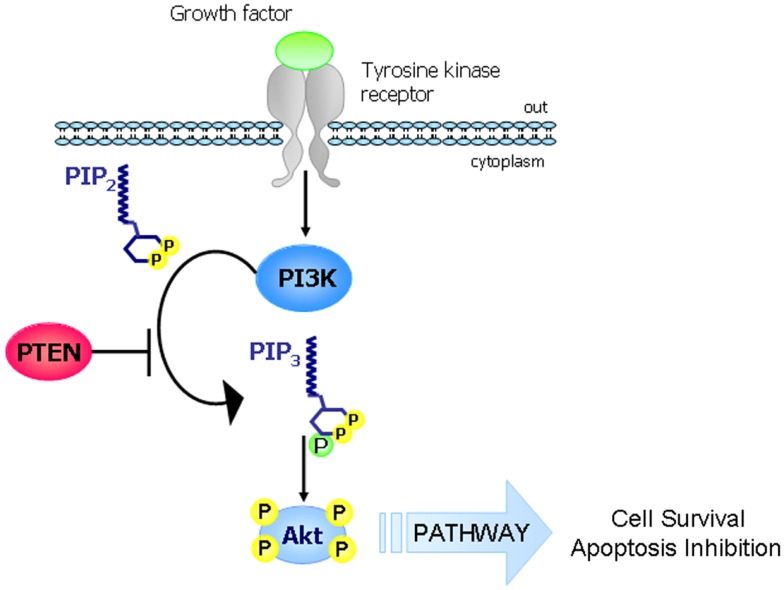
**The PI3K-PTEN-Akt pathway**. The main function of PTEN consists in the regulation of the PI3K/Akt/mTOR. In response to extracellular stimuli (e.g., presence of insulin, growth factors, chemokines), PI3K is activated by tyrosine kinase receptors or G-protein-coupled receptors and it phosphorylates PIP2 to generate PIP3 which in turn phosphorylates and activates Akt. PTEN is a lipid phosphatase that antagonizes the action of PI3K by dephosphorylating PIP3 to generate PIP2 (thus blocking the PI3K signaling cascade).

Phosphatase and TENsin homolog deleted on chromosome 10 (*PTEN*), known also as mutated in multiple advanced cancer 1 (*MMAC1*), is a tumor suppressor gene located at chromosome 10q23.31 and encodes for a 403-amino acid protein that possesses both lipid and protein phosphatase activities. The crystal structure of PTEN revealed two major functional domains (a phosphatase domain and a C2 domain) and three structural regions [a short N-terminal phosphatidylinositol (PI)-4,5-bisphosphate (PIP2) binding domain and a C-terminal tail containing PEST sequences and a PDZ-interaction motif] (Figure 1) (1). The PTEN protein is principally involved in the homeostatic maintenance of PI3K/Akt signaling originating from EGFR activation (or activation of other tyrosine kinase receptors or G-protein-coupled receptors) (Figure 2). Its typical function consists of the dephosphorylation of the lipid-signaling second messenger PI 3,4,5-triphosphate (PIP3), a lipid product of the PI-3-kinase (PI3K) (2), thereby directly antagonizing the PI3K function and blocking therefore the activation of downstream signaling events, including PDK1 (akt) and akt/mammalian target of rapamycin (mTOR). The opposite biochemical reaction is catalyzed by PI3Ks, which are associated with cell growth and cell survival (Figure 2). Thus PTEN, which counteracts PI3Ks activity, is involved in inhibition of cell cycle progression, induction of cell death, modulation of arrest signal, and stimulation of angiogenesis (3).

The lipid phosphatase activity of PTEN is the best-characterized physiological function contributing to the tumor suppressor function of PTEN. As no other redundant and/or compensatory family members have been found, PTEN is the only known lipid phosphatase counteracting the PI3K pathway. It is not surprising that loss of PTEN function, resulting therefore in increased PIP3 and persistent activation of PI3K effectors, has an important impact on multiple aspects of cancer development such as cell proliferation, apoptosis resistance, angiogenesis, metabolism regulation, genomic instability, stem cell self-renewal, cellular senescence, and cell migration and metastasis (4, 5).

In its inactive state, PTEN is phosphorylated on a cluster of serine and threonine residues located on its C-terminal tail, leading to a closed PTEN state and maintaining PTEN protein in a stable conformation. When PTEN is being activated, dephosphorylation of its C-terminal tail opens its phosphatase domain, thereby increasing PTEN activity. Meanwhile, the open state of PTEN is more susceptible to ubiquitin-mediated proteasomal degradation (4, 6): therefore, this mechanism is a negative feed-back leading to decreasing and switching off the effect of PTEN, in absence of specific stimuli.

The functionality of PTEN is also regulated by subcellular localization. PTEN is well characterized as a cytosolic protein that is recruited to the membrane by interacting with a number of membrane-anchored proteins, via its C-terminal PDZ domain and PIP2-binding domain (7). In addition, PTEN mono-ubiquitination controls PTEN nuclear entry. In some tumors, the subcellular localization of PTEN protein seems to mediate its activity (8). The absence of PTEN has been reported to be associated with more aggressive diseases and with high degree of neoplastic transformation, suggesting an important nuclear function for PTEN in tumor suppression (9, 10).

A number of factors have been shown to transcriptionally regulate *PTEN* mRNA [reviewed by Song et al. (5)], including peroxisome proliferation-activated receptor γ (PPARγ), early growth-response protein 1 (EGR1), and p53. *PTEN* mRNA is also post-transcriptionally regulated by *PTEN*-targeting microRNAs such as miR19 and miR21 and is now emerging that also *PTEN* pseudogene (*PTENP1*) may be able to regulate PTEN expression (5).

PTEN loss of function occurs in a wide spectrum of human cancers through various genetic alterations including point mutations (missense and nonsense mutations), large chromosomal deletions (homozygous/heterozygous deletion, frameshift, inframe deletion, and truncation), and epigenetic mechanisms as hypermethylation of the *PTEN* promoter region. In addition, PTEN could be inactivated by other non-structural alterations affecting transcript stability, protein stability, and differential subcellular compartmentalization (4, 5, 8).

Despite its serine, threonine and tyrosine phosphatase activity, the lipid phosphatase function of PTEN has been shown to be the major driving force in tumor suppression. In fact the G129E mutation, observed in cancer specimens and abrogating the lipid phosphatase activity but maintaining its protein phosphatase activity, leads to PTEN tumor suppressor function inactivation *in vitro* (11–13).

Loss of heterozygosity at 10q23 occurs frequently in many sporadic tumors at advanced stage; for example, approximately 70% glioblastoma and 60% advanced prostate cancer are characterized by loss of that region. Somatic mutation in the second allele of *PTEN*, which results in biallelic inactivation, occurs in 25–40% of glioblastomas.

Somatic mutations of *PTEN* have been identified as the main mechanism of inactivation in many tumor types, particularly those of the endometrium, brain, skin, and prostate. The tumor suppressor function of PTEN is usually abrogated following mutations occurring in its phosphatase domain (encoded by exon 5): typically, the C124S mutation (that abrogates both lipidic and protein phosphatase activity) and the G129E mutation (that abrogates only lipid phosphatase activity) (4, 14). Although the N-terminal phosphatase domain is principally responsible for PTEN physiological activity, approximately 40% of *PTEN* tumorigenic mutations may occur in the C-terminal C2 domain (corresponding to exons 6, 7, and 8) and in the tail sequence (corresponding to exon 9), encoding for tyrosine kinase phosphorylation sites important for maintaining PTEN function and protein stability (3, 4, 8, 15). In endometrial carcinoma, glioblastoma, and lymphoma, cancer-specific mutations have been found also in the PIP2-binding region, thus highlighting the importance of this motif for the functionality of PTEN protein (16, 17). In addition to missense mutations, a number of nonsense and frameshift mutations have been described leading to truncated PTEN proteins lacking the C-terminal tail and the PDZ-interaction motif, important domains for PTEN protein stability and recruitment to the membrane, without which PTEN is biochemically inactive (5, 8).

However, in sporadic tumors, loss of heterozygosity of *PTEN* occurs at a much higher frequency than biallelic inactivation. It remains unclear whether haploinsufficiency of PTEN provides a selective growth advantage in tumors lacking a second hit in the remaining *PTEN* allele. Evidence for a role of PTEN haploinsufficiency was demonstrated in a mouse model of prostate cancer in which the dosage of PTEN was inversely correlated to the severity of tumor phenotype (18).

Finally, PTEN can be altered also in inherited syndromes. That is the case of the Cowden disease whose patients tend to develop breast, thyroid, and skin tumors. In these types of tumor PTEN exerts its role in the initiation and in the progression of cancer (3, 12, 19).

## PTEN in Colorectal Cancer

In CRC PTEN is altered through a mixed genetic/epigenetic mechanism (typically: mutations and promoter hypermethylation or 10q23 LOH and promoter hypermethylation), which leads to the biallelic inactivation of the protein in 20–30% of cases.

PTEN expression and mutational rate was reported to be lower in left-sided (distal) CRC in comparison to right-sided (proximal) cancers (20–22). This finding may be related to different genetic mechanisms underlying the tumorigenesis of proximal and distal sporadic CRCs. Cancers arising in right colon are usually characterized by microsatellite instability (MSI), whereas those arising in the distal colon and in the rectum are very often characterized by chromosomal instability (CIN). Therefore, it can be argued that PTEN alterations may be linked to MSI and to the mechanisms leading to MSI (including high frequency of promoter hypermethylation, the main mechanism of mismatch repair genes silencing, whose absence of function is directly responsible of MSI). Consistent with this hypothesis, Day and colleagues found that *PTEN* mutations, identified in about 6% out of 744 stage I-IV CRC, were associated with mucinous histology, MSI, CpG island methylator phenotype, and *BRAF* mutations (22). Furthemore, other reports demonstrated a direct association between *PTEN* mutations and MSI, suggesting that the *PTEN* gene is a target of genomic instability in MSI colorectal tumorigenesis (23–25). In particular, Zhou and colleagues found that among 11 HNPCC CRC, 32 MSI sporadic cancer, and 39 microsatellite stable tumors, *PTEN* somatic mutations were found in 18, 13, and 0% of cases respectively, and PTEN loss of expression (evaluated by IHC) in 31, 41, and 17%, respectively. The majority of somatic mutations occur in the two 6(A) coding mononucleotide tracts, suggesting an etiological role of the deficient mismatch repair system (25). Moreover, it was also reported that *PTEN* promoter hypermethylation is a frequent event in sporadic CRC with MSI and may represent an important epigenetic mechanism of PTEN inactivation in this setting (26).

Overall, although another study did not confirm this association (because gene mutations and LOH were found in about 20 and 17% of sporadic CRC respectively, all but one of which were microsatellite stable) (27), we can assume that PTEN alterations and MSI are correlated.

In addition to PTEN level, the PI3K pathway can be altered following mutations in genes encoding for PI3K proteins, typically in *PIK3CA* gene. Therefore, it has been proposed that PTEN alterations and *PIK3CA* mutations may be mutually exclusive. However, this concept has not been deeply demonstrated as few studies investigating this topic showed conflicting results. There is in fact a clear evidence that mutations in multiple components of the PI3K pathway are not necessarily redundant. Although activating mutations in *PI3K* and loss of PTEN function both enhance PI3K signaling, these alterations seem not to cover equivalent functions. For example, in endometrial cancer, mutations in *PTEN* and *PIK3CA* both occur frequently and often concomitantly within the same tumor, indicating a potential additive or synergistic effect (28–30).

As for the other genetic alterations mainly occurring in CRC, it has been demonstrated that loss of PTEN expression measured by IHC co-occurs with *KRAS* and *BRAF* mutations and with EGFR polysomy (31), whereas *PTEN* and *TP53* mutations seem to be mutually exclusive (27).

## Prognostic Role of PTEN

The role of PTEN as a prognostic factor in CRC has been addressed by relatively few works.

Although accumulating evidence has strongly suggested that PTEN is a crucial factor in various central processes of cancer development, and although in several tumor types (e.g., non-small-cell lung cancer, prostate and breast cancer) PTEN protein status has been correlated with poor prognosis, the association between PTEN expression and clinical parameters in CRC is still controversial. The studies reporting the clinical impact of PTEN alterations on patient outcome in CRC are here summarized. Several of these studies suggest an association between loss of PTEN protein expression with advanced disease, liver metastasis, and poor patient survival, whereas other works do not find such an association (Tables 1 and 2).

**Table 1:** **List of papers finding a positive correlation between PTEN loss and prognosis**.

Author	No.	Type of tissue	Method	% PTEN alteration
Dicuonzo et al. (32)	36	Frozen CRC	Sequencing	17% mutations
Nassif et al. (33)	41	Frozen normal tissue and CRC	Sequencing, LOH, IHC	19% mutations
				17% LOH
				70% reduction or loss of expression (IHC) (cytoplasm and nuclear staining)
Sawai et al. (34)	69 with liver metastasis; 70 without liver metastasis	FFPE CRC and liver metastasis	IHC	75.4% weak expression (cytoplasm and nuclear staining)
Lin et al. (35)	139	FFPE TMA CRC	IHC	7% weak or loss expression (cytoplasm staining)
Li et al. (36)	327	FFPE TMA CRC	Sequencing, IHC	29% weak or loss of expression (PTEN immunoreactivity localized in the nucleus)
Jang et al. (37)	482	FFPE TMA CRC	IHC	50% loss of expression
Jin et al. (38)	68	FFPE CRC	IHC	67.6% loss of expression (cytoplasm and nuclear staining)
Atreya et al. (39)	56	FFPE mCRC	IHC	12.3% loss of expression (cytoplasm and nuclear staining)
Bohn et al. (40)	307	FFPE TMA CRC	FISH	8.8% gene loss

*No.: number of patients; CRC: colorectal cancer; FFPE: formalin-fixed paraffin embedded; FISH: fluorescent *in situ* hybridization; IHC: immunohistochemistry; LOH: loss of heterozygosity; mCRC: metastatic colorectal cancer; TMA: tissue microarray*.

**Table 2:** **List of papers finding no correlation between PTEN loss and prognosis**.

Author	No.	Type of tissue	Method	% PTEN alteration
Colakoglu et al. (21)	76	FFPE CRC	IHC	5% loss of expression; 67% weakly moderate positive expression (cytoplasm staining)
Eklöf et al. (41)	197 and 414*	FFPE CRC	IHC	12.5 and 14% loss of expression (cytoplasm staining)
Price et al. (42)	302	FFPE advanced CRC	Taqman copy number assay	38.7% loss
Day et al. (22)	1093	FFPE stage I-IV CRC	Sequencing	5.8% mutations

*Separate cohort; No.: number of patients; CRC: colorectal cancer; FFPE: formalin-fixed paraffin embedded; IHC: immunohistochemistry.

One of the first paper reporting an association between PTEN alteration and tumor aggressiveness was published in 2001 and examined *PTEN* somatic mutations in a series of 36 sporadic CRC. The authors found that *PTEN* gene mutations were detected only in patients with locally advanced or metastatic CRC (32).

The majority of the next studies have been performed by analyzing PTEN protein expression by IHC assay, the most effective way to assess the loss of PTEN function by any mechanism (LOH, somatic mutation, or promoter epigenetic silencing). In fact, it has been reported that all tumors with *PTEN* gene alterations (mutation and/or deletion) showed a reduction or absence of PTEN expression evaluated by IHC, and this finding was correlated with advanced stage of disease (33). This association was confirmed by Sawai and colleagues, who demonstrated that PTEN loss was significantly correlated with local recurrence, advanced TNM stage (*p* < 0.01), lymph node metastasis (*p* < 0.05) and with lower 5-year survival rate (*p* = 0.012), indicating a link between PTEN deregulation and CRC aggressive phenotype (34). A positive association of PTEN expression with histological grade and distant metastasis was also demonstrated by Lin and colleagues (35). Similarly, Li and co-workers, by examining nuclear PTEN protein expression on tissue microarray in 327 CRC, found that low level of PTEN protein expression was positively correlated with tumor size, depth of invasion, lymphatic invasion, lymph node metastasis, and higher tumor staging (*p* < 0.05). In addition, univariate and multivariate analysis indicated that patients characterized by PTEN loss of protein expression had a shorter survival than patients with a normal expression of PTEN (36).

Another study performed on 482 CRC revealed that PTEN protein expression (evaluated again on a tissue microarray) was associated with poor overall survival (OS) and disease-free survival (*p* = 0.03 and *p* = 0.046, respectively), although in multivariate analysis, a significant difference was observed only in patients with stage II of disease (37).

Jin and colleagues by evaluating the prognostic value of PTEN, STAT3, and VEGF-C protein expression by IHC in 68 cases of CRC, showed that PTEN expression was correlated with pathological grade, but not with tumor size, lymph node metastasis, or clinical stage. Moreover the 3- and 5-years survival rates of patients normally expressing PTEN were significantly higher than those of patients with a PTEN-negative tumor (38).

In a very recent study conducted on 56 patients affected by a metastatic disease, PTEN protein expression was analyzed by an optimized PTEN IHC assay recently developed and it was found that the median OS of patients whose tumors did not express PTEN was 9 months, compared to 49 months for patients with a normal expression of PTEN [HR = 6.25, 95% confidence intervals (CI), *p* = 0.0023]. The association of absence of PTEN expression with increased risk of death remained significant in multivariate analysis (Hazard Ratio, HR = 6.31, 95% CI, *p* = 0.0023) (39).

Finally, the positive correlation between worse prognosis and PTEN alteration was also found after the analysis of genetic lesions. Through the evaluation of *PTEN* deletion and gene rearrangements by FISH on 307 CRC, the authors confirmed an association between *PTEN* alteration with reduced patient survival in univariate and multivariate analyses in rectal cancer (*p* = 0.012, HR 2.675; 95% CI) but not in colon cancer (40).

On the contrary with respect to the results obtained by the studies reported above, Colakoglu and colleagues, by investigating 76 CRC patients, found no correlation between PTEN immunohistochemical status and patient survival, tumor grade, TNM stage, lymphatic invasion, and liver metastasis (21), although they found a significant association between PTEN loss and local recurrence. Another study investigating the prognostic role of *KRAS, BRAF, PIK3CA* mutations, and PTEN expression in two separate CRC cohorts of 197 and 414 patients respectively, observed absence of correlation between PTEN status and prognosis by analyzing each molecular marker separately (41). The prognostic value of PTEN was also explored through the evaluation of *PTEN* gene copy number alteration (CNA) assessed by a Taqman assay by Price and colleagues in a cohort of 302 patients with advanced CRC enrolled in the AGITG MAX trial, a randomized Phase III trial of capecitabine ± bevacizumab or mitomycin C. The authors did not find any correlation between *PTEN* status and progression free survival (PFS) or OS in multivariate analysis (42). The absence of association with prognosis in stage II and III CRC was also supported by the work of Day and colleagues who analyzed *PTEN* mutations in a large cohort of sporadic CRC (22).

In conclusion, at the moment there are no clinical data clearly supporting the notion of PTEN alteration as a prognostic factor in CRC.

## Predictive Role of PTEN in EGFR-Targeted Therapies Response

In addition to the evaluation of the prognostic role of PTEN, several studies have investigated its predictive role in the field of targeted therapies. Since in breast cancer patients it has been demonstrated that PTEN loss of expression confers resistance to trastuzumab (a monoclonal antibody, MoAb, against Her-2, a tyrosine kinase receptor belonging to the Her family, as EGFR) (43), recent reports have investigated whether PTEN alterations may affect responsiveness of mCRC patients to anti-EGFR MoAbs cetuximab and panitumumab (Table 3). These studies have primarily used IHC to assess expression at protein level, and some have shown a correlation between PTEN expression and clinical response. A preliminary work on a retrospective series of patients reported that loss of PTEN expression, observed in 40% of primary tumor in mCRC patients, was significantly associated with non-responsiveness to cetuximab (44). The authors found that no patients with PTEN loss of expression in tumor tissue responded to a combination of irinotecan and cetuximab, whereas 10 out of 16 (63%) patients with intact PTEN expression experienced a partial response to these therapies. *In vitro* studies have confirmed this evidence by showing that *PIK3CA* mutations or PTEN loss may predict the efficacy of cetuximab administration in colon cancer cell lines (45). The role of PTEN in predicting resistance to anti-EGFR MoAbs was confirmed by Sartore-Bianchi and colleagues, who found that loss of PTEN protein was associated with lack of response to cetuximab and panitumumab (*p* = 0.001) in a cohort of 81 tumor specimens. Loss of PTEN expression was also associated with shorter PFS and worse OS (46). Supporting these data, another study showed that inactivation of PTEN protein by gene mutation or deletion (detected by FISH) was responsible of cetuximab resistance (47). Razis and colleagues, did not find any association between PTEN protein expression as evaluated by IHC with clinical outcomes, although the lack of *PTEN* gene amplification evaluated by FISH was associated with a better response rate and longer time to progression (48).

**Table 3:** **List of papers investigating the predictive role of PTEN in CRC treated with EGFR-targeted therapies cetuximab or panitumumab**.

Author	No.	Type of tissue	Method	% PTEN alteration and clinical response
Frattini et al. (44)	27	FFPE mCRC	IHC	100% PTEN-negative patients were NR (*p* < 0.001)
Sartore-Bianchi et al. (46)	81	FFPE mCRC	IHC	97% PTEN-negative patients were NR (*p* = 0.001)
Perrone et al. (47)	32	FFPE mCRC	Sequencing, FISH	All patients with a decreased *PTEN* gene copy number or with PTEN mutation were NR
Razis et al. (48)	72	FFPE mCRC	IHC and FISH	PTEN gene deletion detected only by FISH associated with no response
Loupakis et al. (49)	102	FFPE mCRC (primary and metastatic lesion)	IHC	95% PTEN-negative patients were NR. Association with clinical response found only in the metastatic lesion
Negri et al. (50)	50	FFPE mCRC (primary and metastatic lesion)	Immunofluorescence	100% PTEN-negative patients were NR (*p* < 0.05). Association with clinical response found only in the metastatic lesion
Tol et al. (51)	559	FFPE mCRC	IHC	Loss of PTEN expression observed in 42% but not associated with response
Ulivi et al. (52)	67	FFPE mCRC	IHC	Loss of PTEN expression observed in 60% but not associated with response
Laurent-Puig et al. (53)	162	FFPE mCRC	IHC	Loss of PTEN expression observed in 19% but not associated with response

*No: number of patients; CRC: colorectal cancer; FFPE: formalin-fixed paraffin embedded; FISH: fluorescent *in situ* hybridization; IHC: immunohistochemistry; mCRC: metastatic colorectal cancer; NR: non-responder to anti-EGFR therapies*.

A substantial but not complete confirmation of these data has been reported by Loupakis and colleagues which demonstrated that loss of PTEN expression was not associated with resistance to cetuximab plus irinotecan in the primary tumor (*n* = 96), but was associated with lack of response in the metastatic lesion (the analysis was performed in 59 cases). In the PTEN-positive group, 12 out of 33 (36%) patients benefited from the therapy whereas only 1 patient out of 22 (5%) cases with a PTEN-negative profile responded to EGFR-targeted drugs (*p* = 0.007). Moreover, patients with PTEN-positive metastases and *KRAS* wild-type gene sequence had longer PFS compared with other patients (49). According to these data, Negri and colleagues evaluated PTEN expression by immunofluorescence both in primary and metastatic sites in CRC patients treated with cetuximab and they found that the loss of PTEN expression in metastatic sites was negatively associated with response (50).

On the contrary with respect to the previous works, other studies failed to demonstrate a correlation between loss of PTEN expression and response to anti-EGFR MoAbs. In a large cohort of 559 mCRC patients treated with chemotherapy and bevacizumab with or without cetuximab (phase III CAIRO2 study), the authors did not find any correlation between PTEN loss evaluated by IHC and response to treatment with cetuximab, neither individually nor in combination with other markers (51). This result was confirmed by Ulivi et al. by the analysis of 67 mCRC patients receiving cetuximab (52). Finally, the investigation of 162 samples by Laurent-Puig and colleagues reported the PTEN null expression rate of 19.9% with an association of poorer OS in the *KRAS* wild-type population (*p* = 0.013) but not with tumor response or PFS, thus suggesting the PTEN loss of expression as a prognostic rather than a predictive role (53).

## Conclusion

According to the reported results, the role played by PTEN as a prognostic or predictive marker in CRC is still a matter of debate. Discordant results have been reported and this fact could be attributable to several issues: (1) the size of the analyzed cohort, (2) patients inclusion criteria, (3) the methods of assessing PTEN alteration. For the latter point, it should be noted that the majority of studies have evaluated PTEN alteration by IHC, the easier and cheaper method to be used. However, these studies showed highly discordant results, with PTEN loss ranging from 5% up to 66% (40). Reasons for this variability might include inherent issues with IHC. Interpreting PTEN data can be challenging, because immunohistochemistry can produce variable results. The lack of standardized methods and the variability of tissue handling may bias the PTEN expression analysis by IHC. In addition, IHC is afflicted by intra and inter-observer variability due to the semi-quantitative character of such an analysis. A standard, universally accepted PTEN testing and scoring system for PTEN IHC evaluation, has yet to be established. Assessment of PTEN expression is further complicated by potential discordance between the expression of PTEN in the primary and in the metastatic tissue. Concordance rates vary from 47 to 98% between primary and metastatic lesions (39, 49, 54–56). These differences may impair the prediction of anti-EGFR therapies outcome. Loupakis and colleagues reported in fact that PTEN loss was predictive of cetuximab resistance only by evaluating the metastatic lesion (49). Sangale and co-workers however, has recently developed an optimized PTEN IHC assay developed through a rigorous testing of antibody specificity and selectivity using samples with known molecular alterations in PTEN, paired with reproducible method of interpretation. The Authors found a 98% of concordance of PTEN expression between primary and metastatic tumors (57). Another issue that has recently emerged is the intracellular localization of PTEN protein. Some researchers demonstrated that PTEN is localized both in the cytoplasm and into the nucleus and shuttles between these two compartments can be influenced by a variety of mechanisms. Accumulating genetic, pathologic and biochemical evidence suggests that the localization of PTEN either in the nucleus or cytoplasm may affect the proliferation of tumor cells (58–60). Another point that could affect the establishment of the prognostic and predictive value of PTEN is haploinsufficiency, determined when only one allele is altered, as it remains unclear whether this condition could provide a selective growth advantage in tumors lacking a second hit in the remaining *PTEN* allele.

To clarify the problems concerning PTEN evaluation, it would be necessary a comparison of the results obtained by analyzing the several PTEN alterations through different methodologies (FISH, promoter methylation, LOH, and immunohistochemistry performed with different antibodies) both on cancer cell lines with a well known PTEN status and on a large series of patients in order to better establish IHC evaluation criteria. An international interlaboratory reproducibility ring study [as that performed for EGFR FISH analysis in metastatic colorectal cancer (CRC) patients] (61) is missing and has to be performed in order to ascertain the difficulties and the discrepancies in PTEN evaluations in different laboratories.

In conclusion, much work, especially in large and homogeneous cohorts of cases from different laboratories, has to be done before the establishment of PTEN as prognostic or predictive marker in CRC. On the contrary, in other tumor types (such as breast cancer), this role is clearer.

## Conflict of Interest Statement

The authors declare that the research was conducted in the absence of any commercial or financial relationships that could be construed as a potential conflict of interest.
